# *n*-Undeca­nyl 2-(4-bromo­anilino)-4,4-dimethyl-6-oxocyclo­hex-1-ene­carbodithio­ate

**DOI:** 10.1107/S1600536809006175

**Published:** 2009-02-25

**Authors:** El Sayed H. El Ashry, Mohammed R. Amer, M. Raza Shah, Seik Weng Ng

**Affiliations:** a H.E.J. Research Institute of Chemistry, International Center for Chemical and Biological Sciences, University of Karachi, Karachi 75270, Pakistan; b Department of Chemistry, University of Malaya, 50603 Kuala Lumpur, Malaysia

## Abstract

The six-membered cyclo­hexene ring in the title compound, C_26_H_38_BrNOS_2_, adopts an envelope conformation, with the C atom bearing the two methyl groups representing the flap. This atom deviates by 0.651 (3) Å from the plane passing through the other five atoms of the ring (r.m.s. deviation = 0.051 Å). The mol­ecular conformation is stabilized by an N—H⋯S hydrogen bond. The title compound is isomorphous with *n*-undeca­nyl 2-(4-chloro­anilino)-4,4-dimethyl-6-oxocyclo­hex-1-enecarbodithio­ate.

## Related literature

For background, see: El Ashry *et al.* (2009*a* ▶). For the isostructural *n*-undeca­nyl 2-(4-chloro­anilino)-4,4-dimethyl-6-oxocyclo­hex-1-enylcarbodithio­ate, see: El Ashry *et al.* (2009*b* ▶).
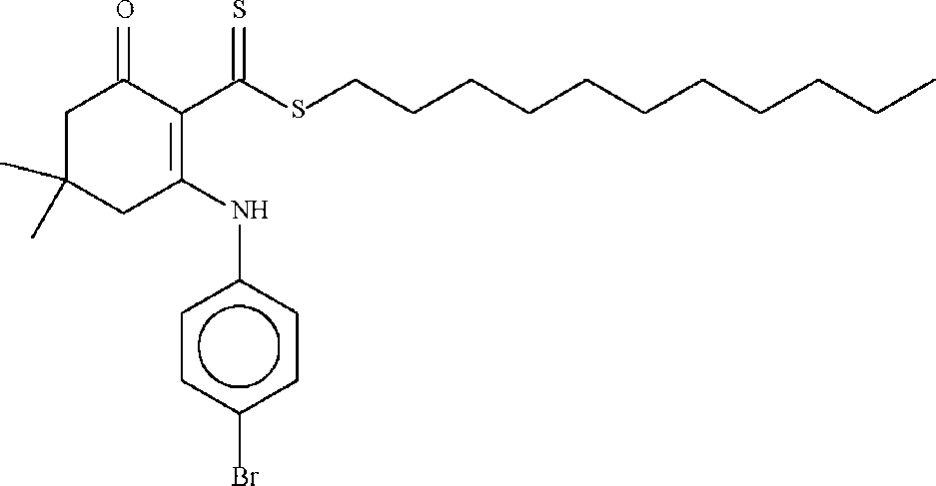


## Experimental

### 

#### Crystal data

C_26_H_38_BrNOS_2_
*M* *_r_* = 524.60
Triclinic, 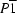

*a* = 8.0469 (2) Å
*b* = 11.8346 (3) Å
*c* = 14.9374 (3) Å
α = 95.863 (1)°
β = 95.414 (2)°
γ = 106.595 (1)°
*V* = 1344.70 (5) Å^3^
*Z* = 2
Mo *K*α radiation
μ = 1.70 mm^−1^
*T* = 100 K
0.45 × 0.15 × 0.05 mm

#### Data collection

Bruker SMART APEX diffractometer
Absorption correction: multi-scan (*SADABS*; Sheldrick, 1996 ▶) *T*_min_ = 0.768, *T*_max_ = 0.920
12608 measured reflections
6149 independent reflections
4426 reflections with *I* > 2σ(*I*)
*R*_int_ = 0.028

#### Refinement

*R*[*F* ^2^ > 2σ(*F* ^2^)] = 0.036
*wR*(*F* ^2^) = 0.104
*S* = 1.02
6149 reflections
287 parameters
1 restraint
H atoms treated by a mixture of independent and constrained refinement
Δρ_max_ = 0.60 e Å^−3^
Δρ_min_ = −0.74 e Å^−3^

### 

Data collection: *APEX2* (Bruker, 2008 ▶); cell refinement: *SAINT* (Bruker, 2008 ▶); data reduction: *SAINT*; program(s) used to solve structure: *SHELXS97* (Sheldrick, 2008 ▶); program(s) used to refine structure: *SHELXL97* (Sheldrick, 2008 ▶); molecular graphics: *X-SEED* (Barbour, 2001 ▶); software used to prepare material for publication: *publCIF* (Westrip, 2009 ▶).

## Supplementary Material

Crystal structure: contains datablocks global, I. DOI: 10.1107/S1600536809006175/bt2879sup1.cif

Structure factors: contains datablocks I. DOI: 10.1107/S1600536809006175/bt2879Isup2.hkl

Additional supplementary materials:  crystallographic information; 3D view; checkCIF report

## Figures and Tables

**Table 1:** Hydrogen-bond geometry (Å, °)

*D*—H⋯*A*	*D*—H	H⋯*A*	*D*⋯*A*	*D*—H⋯*A*
N1—H1⋯S2	0.88 (1)	2.105 (19)	2.876 (2)	146 (3)

**Fig. 1.**
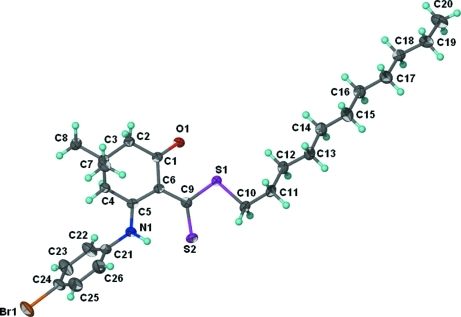
Anisotropic displacement ellipsoid plot (Barbour, 2001) plot of C26H38BrNOS2 at the 70% probability level; hydrogen atoms are drawn as spheres of arbitrary radius.

## supplementary crystallographic information

### Experimental

To a solution of (4-bromophenylamino)-5,5-dimethyl-cyclohex-2-en-1-one (0.1 mol) in DMSO (20 ml) and sodium hydroxide (0.4 g) in water (1 ml), carbon disulfide (0.3 mol) was added in the course of 30 minutes. The mixture was stirred for 20 min at 283 K, and then 1-bromoundecane (0.1 mol) was added drop wise at room temperature for 30 min. The reaction mixture was left for 24 h and then diluted with water (200 ml) and acidified with 10% hydrochloric acid. The resulting precipitate was collected by filtration, dried and purified on silica gel column (40% ethyl acetate in hexane) to give yellow crystal (38% yield; mp.410 K).

### Refinement

Carbon-bound H-atoms were placed in calculated positions (C—H 0.93 to 0.99 Å) and were included in the refinement in the riding model approximation, with *U*(H) set to 1.2 to 1.5*U*(C). The methyl groups were allowed to rotate but not to tip. The amino H-atom was located in a difference Fourier map, and was refined with a distance restraint of N–H 0.88±0.01 Å; its isotropic displacement parameter was freely refined.

### Crystal data

**Table:**

C_26_H_38_BrNOS_2_	*Z* = 2
*M**_r_* = 524.60	*F*(000) = 552
Triclinic, *P*1	*D*_x_ = 1.296 Mg m^−^^3^
Hall symbol: -P 1	Mo *K*α radiation, λ = 0.71073 Å
*a* = 8.0469 (2) Å	Cell parameters from 3369 reflections
*b* = 11.8346 (3) Å	θ = 2.7–27.3°
*c* = 14.9374 (3) Å	µ = 1.70 mm^−^^1^
α = 95.863 (1)°	*T* = 100 K
β = 95.414 (2)°	Chip, orange
γ = 106.595 (1)°	0.45 × 0.15 × 0.05 mm
*V* = 1344.70 (5) Å^3^	

### Data collection

**Table:**

Bruker SMART APEX diffractometer	6149 independent reflections
Radiation source: fine-focus sealed tube	4426 reflections with *I* > 2σ(*I*)
graphite	*R*_int_ = 0.028
ω scans	θ_max_ = 27.5°, θ_min_ = 1.4°
Absorption correction: multi-scan (*SADABS*; Sheldrick, 1996)	*h* = −10→10
*T*_min_ = 0.768, *T*_max_ = 0.920	*k* = −15→15
12608 measured reflections	*l* = −18→19

### Refinement

**Table:**

Refinement on *F*^2^	Primary atom site location: structure-invariant direct methods
Least-squares matrix: full	Secondary atom site location: difference Fourier map
*R*[*F*^2^ > 2σ(*F*^2^)] = 0.036	Hydrogen site location: inferred from neighbouring sites
*wR*(*F*^2^) = 0.104	H atoms treated by a mixture of independent and constrained refinement
*S* = 1.02	*w* = 1/[σ^2^(*F*_o_^2^) + (0.0482*P*)^2^ + 0.562*P*] where *P* = (*F*_o_^2^ + 2*F*_c_^2^)/3
6149 reflections	(Δ/σ)_max_ = 0.001
287 parameters	Δρ_max_ = 0.60 e Å^−^^3^
1 restraint	Δρ_min_ = −0.73 e Å^−^^3^

### Fractional atomic coordinates and isotropic or equivalent isotropic displacement parameters (Å^2^)

**Table:**

	*x*	*y*	*z*	*U*_iso_*/*U*_eq_	
Br1	0.20125 (4)	0.40758 (3)	1.043527 (19)	0.04841 (12)	
S1	0.63664 (8)	0.25017 (5)	0.39673 (4)	0.01981 (14)	
S2	0.39769 (8)	0.15843 (5)	0.52739 (4)	0.02570 (15)	
N1	0.4559 (3)	0.34432 (19)	0.67717 (14)	0.0227 (5)	
H1	0.417 (4)	0.2707 (13)	0.6491 (19)	0.043 (9)*	
O1	0.7716 (2)	0.47491 (15)	0.43470 (11)	0.0243 (4)	
C1	0.7367 (3)	0.4886 (2)	0.51245 (15)	0.0176 (5)	
C2	0.8229 (3)	0.6079 (2)	0.56838 (15)	0.0197 (5)	
H2A	0.7669	0.6658	0.5459	0.024*	
H2B	0.9471	0.6343	0.5580	0.024*	
C3	0.8164 (3)	0.6126 (2)	0.67035 (16)	0.0196 (5)	
C4	0.6285 (3)	0.5481 (2)	0.68353 (16)	0.0194 (5)	
H4A	0.6216	0.5463	0.7492	0.023*	
H4B	0.5510	0.5936	0.6612	0.023*	
C5	0.5629 (3)	0.4234 (2)	0.63549 (15)	0.0171 (5)	
C6	0.6129 (3)	0.3925 (2)	0.54951 (15)	0.0172 (5)	
C7	0.9431 (3)	0.5538 (2)	0.71398 (17)	0.0267 (6)	
H7A	1.0631	0.5981	0.7068	0.040*	
H7B	0.9162	0.4717	0.6845	0.040*	
H7C	0.9316	0.5539	0.7787	0.040*	
C8	0.8613 (3)	0.7423 (2)	0.71367 (18)	0.0287 (6)	
H8A	0.7759	0.7784	0.6875	0.043*	
H8B	0.9786	0.7862	0.7020	0.043*	
H8C	0.8583	0.7453	0.7793	0.043*	
C9	0.5497 (3)	0.2747 (2)	0.49803 (16)	0.0190 (5)	
C10	0.5389 (3)	0.0919 (2)	0.36124 (17)	0.0244 (5)	
H10A	0.5476	0.0477	0.4134	0.029*	
H10B	0.4137	0.0750	0.3380	0.029*	
C11	0.6358 (3)	0.0527 (2)	0.28703 (16)	0.0239 (5)	
H11A	0.7599	0.0693	0.3121	0.029*	
H11B	0.5864	−0.0344	0.2699	0.029*	
C12	0.6282 (3)	0.1122 (2)	0.20152 (16)	0.0257 (6)	
H12A	0.5043	0.0984	0.1771	0.031*	
H12B	0.6827	0.1991	0.2176	0.031*	
C13	0.7204 (3)	0.0665 (2)	0.12828 (17)	0.0269 (6)	
H13A	0.6626	−0.0198	0.1105	0.032*	
H13B	0.8429	0.0771	0.1537	0.032*	
C14	0.7206 (4)	0.1286 (2)	0.04413 (17)	0.0285 (6)	
H14A	0.5981	0.1159	0.0176	0.034*	
H14B	0.7748	0.2153	0.0622	0.034*	
C15	0.8177 (4)	0.0858 (3)	−0.02797 (17)	0.0303 (6)	
H15A	0.7686	−0.0016	−0.0433	0.036*	
H15B	0.9421	0.1036	−0.0029	0.036*	
C16	0.8069 (4)	0.1428 (2)	−0.11419 (17)	0.0283 (6)	
H16A	0.8577	0.2301	−0.0989	0.034*	
H16B	0.6822	0.1263	−0.1384	0.034*	
C17	0.9010 (4)	0.0991 (3)	−0.18796 (17)	0.0292 (6)	
H17A	1.0267	0.1187	−0.1648	0.035*	
H17B	0.8537	0.0114	−0.2016	0.035*	
C18	0.8831 (3)	0.1527 (2)	−0.27507 (17)	0.0275 (6)	
H18A	0.7572	0.1372	−0.2961	0.033*	
H18B	0.9363	0.2401	−0.2618	0.033*	
C19	0.9672 (3)	0.1054 (3)	−0.35106 (17)	0.0289 (6)	
H19A	0.9186	0.0176	−0.3625	0.035*	
H19B	1.0944	0.1251	−0.3317	0.035*	
C20	0.9383 (4)	0.1558 (3)	−0.4385 (2)	0.0415 (7)	
H20A	0.9946	0.1220	−0.4853	0.062*	
H20B	0.9890	0.2426	−0.4282	0.062*	
H20C	0.8126	0.1355	−0.4586	0.062*	
C21	0.3982 (3)	0.3639 (2)	0.76329 (16)	0.0223 (5)	
C22	0.2948 (3)	0.4369 (3)	0.77828 (18)	0.0321 (6)	
H22	0.2631	0.4780	0.7313	0.039*	
C23	0.2372 (4)	0.4502 (3)	0.86191 (18)	0.0358 (7)	
H23	0.1667	0.5011	0.8728	0.043*	
C24	0.2825 (3)	0.3895 (3)	0.92928 (17)	0.0302 (6)	
C25	0.3827 (4)	0.3158 (3)	0.91502 (19)	0.0395 (7)	
H25	0.4117	0.2735	0.9619	0.047*	
C26	0.4422 (4)	0.3029 (2)	0.83136 (18)	0.0342 (7)	
H26	0.5132	0.2522	0.8210	0.041*	

### Atomic displacement parameters (Å^2^)

**Table:**

	*U*^11^	*U*^22^	*U*^33^	*U*^12^	*U*^13^	*U*^23^
Br1	0.0475 (2)	0.0680 (3)	0.02154 (15)	0.00315 (17)	0.01392 (13)	0.00077 (14)
S1	0.0220 (3)	0.0169 (3)	0.0192 (3)	0.0037 (2)	0.0035 (2)	0.0015 (2)
S2	0.0276 (3)	0.0168 (3)	0.0307 (3)	0.0012 (3)	0.0111 (3)	0.0029 (3)
N1	0.0266 (11)	0.0189 (11)	0.0226 (11)	0.0046 (9)	0.0089 (9)	0.0035 (9)
O1	0.0282 (9)	0.0220 (9)	0.0202 (9)	0.0019 (8)	0.0076 (7)	0.0037 (7)
C1	0.0145 (11)	0.0183 (12)	0.0199 (12)	0.0048 (9)	0.0012 (9)	0.0028 (9)
C2	0.0210 (12)	0.0176 (12)	0.0196 (12)	0.0035 (10)	0.0021 (9)	0.0046 (9)
C3	0.0167 (11)	0.0206 (12)	0.0199 (12)	0.0038 (10)	0.0016 (9)	0.0015 (10)
C4	0.0197 (12)	0.0206 (12)	0.0181 (11)	0.0052 (10)	0.0039 (9)	0.0039 (9)
C5	0.0130 (11)	0.0190 (12)	0.0203 (11)	0.0060 (9)	0.0004 (9)	0.0052 (9)
C6	0.0166 (11)	0.0161 (12)	0.0188 (11)	0.0042 (9)	0.0017 (9)	0.0045 (9)
C7	0.0203 (12)	0.0363 (15)	0.0223 (13)	0.0068 (11)	0.0011 (10)	0.0052 (11)
C8	0.0284 (14)	0.0232 (14)	0.0290 (14)	0.0002 (11)	0.0056 (11)	−0.0017 (11)
C9	0.0181 (11)	0.0215 (12)	0.0191 (11)	0.0080 (10)	0.0020 (9)	0.0051 (10)
C10	0.0287 (13)	0.0166 (12)	0.0244 (13)	0.0024 (11)	0.0029 (10)	0.0001 (10)
C11	0.0282 (13)	0.0164 (12)	0.0252 (13)	0.0055 (11)	0.0023 (10)	−0.0015 (10)
C12	0.0285 (14)	0.0245 (13)	0.0217 (13)	0.0062 (11)	0.0009 (10)	−0.0014 (10)
C13	0.0279 (14)	0.0251 (14)	0.0242 (13)	0.0050 (11)	0.0006 (10)	−0.0018 (11)
C14	0.0322 (14)	0.0279 (14)	0.0247 (13)	0.0093 (12)	0.0025 (11)	0.0011 (11)
C15	0.0319 (15)	0.0364 (16)	0.0232 (14)	0.0118 (13)	0.0024 (11)	0.0028 (12)
C16	0.0295 (14)	0.0286 (14)	0.0263 (14)	0.0084 (12)	0.0031 (11)	0.0016 (11)
C17	0.0281 (14)	0.0340 (15)	0.0247 (13)	0.0076 (12)	0.0029 (11)	0.0051 (11)
C18	0.0272 (13)	0.0244 (14)	0.0287 (14)	0.0039 (11)	0.0044 (11)	0.0042 (11)
C19	0.0242 (13)	0.0350 (15)	0.0283 (14)	0.0080 (12)	0.0050 (11)	0.0093 (12)
C20	0.0422 (17)	0.052 (2)	0.0341 (16)	0.0140 (15)	0.0125 (13)	0.0174 (14)
C21	0.0205 (12)	0.0229 (13)	0.0200 (12)	0.0001 (10)	0.0048 (10)	0.0025 (10)
C22	0.0301 (14)	0.0501 (18)	0.0248 (14)	0.0212 (14)	0.0083 (11)	0.0132 (13)
C23	0.0282 (14)	0.058 (2)	0.0278 (14)	0.0222 (14)	0.0086 (12)	0.0060 (14)
C24	0.0271 (14)	0.0384 (16)	0.0191 (13)	−0.0008 (12)	0.0069 (10)	0.0032 (11)
C25	0.060 (2)	0.0362 (17)	0.0251 (14)	0.0145 (16)	0.0092 (14)	0.0122 (13)
C26	0.0531 (18)	0.0269 (15)	0.0288 (15)	0.0181 (14)	0.0102 (13)	0.0094 (12)

### Geometric parameters (Å, °)

**Table:**

Br1—C24	1.898 (3)	C12—H12B	0.9900
S1—C9	1.758 (2)	C13—C14	1.520 (4)
S1—C10	1.813 (2)	C13—H13A	0.9900
S2—C9	1.687 (2)	C13—H13B	0.9900
N1—C5	1.328 (3)	C14—C15	1.517 (4)
N1—C21	1.427 (3)	C14—H14A	0.9900
N1—H1	0.882 (10)	C14—H14B	0.9900
O1—C1	1.226 (3)	C15—C16	1.520 (4)
C1—C6	1.475 (3)	C15—H15A	0.9900
C1—C2	1.505 (3)	C15—H15B	0.9900
C2—C3	1.525 (3)	C16—C17	1.523 (4)
C2—H2A	0.9900	C16—H16A	0.9900
C2—H2B	0.9900	C16—H16B	0.9900
C3—C7	1.524 (3)	C17—C18	1.517 (4)
C3—C4	1.529 (3)	C17—H17A	0.9900
C3—C8	1.530 (3)	C17—H17B	0.9900
C4—C5	1.496 (3)	C18—C19	1.514 (4)
C4—H4A	0.9900	C18—H18A	0.9900
C4—H4B	0.9900	C18—H18B	0.9900
C5—C6	1.425 (3)	C19—C20	1.516 (4)
C6—C9	1.447 (3)	C19—H19A	0.9900
C7—H7A	0.9800	C19—H19B	0.9900
C7—H7B	0.9800	C20—H20A	0.9800
C7—H7C	0.9800	C20—H20B	0.9800
C8—H8A	0.9800	C20—H20C	0.9800
C8—H8B	0.9800	C21—C22	1.376 (4)
C8—H8C	0.9800	C21—C26	1.381 (4)
C10—C11	1.521 (3)	C22—C23	1.382 (4)
C10—H10A	0.9900	C22—H22	0.9500
C10—H10B	0.9900	C23—C24	1.375 (4)
C11—C12	1.524 (3)	C23—H23	0.9500
C11—H11A	0.9900	C24—C25	1.361 (4)
C11—H11B	0.9900	C25—C26	1.389 (4)
C12—C13	1.517 (3)	C25—H25	0.9500
C12—H12A	0.9900	C26—H26	0.9500
			
C9—S1—C10	103.12 (11)	C12—C13—H13A	108.8
C5—N1—C21	127.6 (2)	C14—C13—H13A	108.8
C5—N1—H1	117 (2)	C12—C13—H13B	108.8
C21—N1—H1	116 (2)	C14—C13—H13B	108.8
O1—C1—C6	121.4 (2)	H13A—C13—H13B	107.7
O1—C1—C2	117.4 (2)	C15—C14—C13	113.7 (2)
C6—C1—C2	121.1 (2)	C15—C14—H14A	108.8
C1—C2—C3	115.96 (19)	C13—C14—H14A	108.8
C1—C2—H2A	108.3	C15—C14—H14B	108.8
C3—C2—H2A	108.3	C13—C14—H14B	108.8
C1—C2—H2B	108.3	H14A—C14—H14B	107.7
C3—C2—H2B	108.3	C14—C15—C16	113.2 (2)
H2A—C2—H2B	107.4	C14—C15—H15A	108.9
C2—C3—C7	111.1 (2)	C16—C15—H15A	108.9
C2—C3—C4	107.11 (19)	C14—C15—H15B	108.9
C7—C3—C4	110.6 (2)	C16—C15—H15B	108.9
C2—C3—C8	109.5 (2)	H15A—C15—H15B	107.7
C7—C3—C8	109.9 (2)	C15—C16—C17	113.9 (2)
C4—C3—C8	108.6 (2)	C15—C16—H16A	108.8
C5—C4—C3	113.52 (19)	C17—C16—H16A	108.8
C5—C4—H4A	108.9	C15—C16—H16B	108.8
C3—C4—H4A	108.9	C17—C16—H16B	108.8
C5—C4—H4B	108.9	H16A—C16—H16B	107.7
C3—C4—H4B	108.9	C18—C17—C16	113.4 (2)
H4A—C4—H4B	107.7	C18—C17—H17A	108.9
N1—C5—C6	122.4 (2)	C16—C17—H17A	108.9
N1—C5—C4	116.4 (2)	C18—C17—H17B	108.9
C6—C5—C4	121.3 (2)	C16—C17—H17B	108.9
C5—C6—C9	124.1 (2)	H17A—C17—H17B	107.7
C5—C6—C1	116.5 (2)	C19—C18—C17	114.3 (2)
C9—C6—C1	119.4 (2)	C19—C18—H18A	108.7
C3—C7—H7A	109.5	C17—C18—H18A	108.7
C3—C7—H7B	109.5	C19—C18—H18B	108.7
H7A—C7—H7B	109.5	C17—C18—H18B	108.7
C3—C7—H7C	109.5	H18A—C18—H18B	107.6
H7A—C7—H7C	109.5	C18—C19—C20	112.9 (2)
H7B—C7—H7C	109.5	C18—C19—H19A	109.0
C3—C8—H8A	109.5	C20—C19—H19A	109.0
C3—C8—H8B	109.5	C18—C19—H19B	109.0
H8A—C8—H8B	109.5	C20—C19—H19B	109.0
C3—C8—H8C	109.5	H19A—C19—H19B	107.8
H8A—C8—H8C	109.5	C19—C20—H20A	109.5
H8B—C8—H8C	109.5	C19—C20—H20B	109.5
C6—C9—S2	125.08 (18)	H20A—C20—H20B	109.5
C6—C9—S1	117.86 (17)	C19—C20—H20C	109.5
S2—C9—S1	117.06 (14)	H20A—C20—H20C	109.5
C11—C10—S1	108.50 (16)	H20B—C20—H20C	109.5
C11—C10—H10A	110.0	C22—C21—C26	120.0 (2)
S1—C10—H10A	110.0	C22—C21—N1	121.8 (2)
C11—C10—H10B	110.0	C26—C21—N1	118.1 (2)
S1—C10—H10B	110.0	C21—C22—C23	119.9 (3)
H10A—C10—H10B	108.4	C21—C22—H22	120.1
C12—C11—C10	115.1 (2)	C23—C22—H22	120.1
C12—C11—H11A	108.5	C24—C23—C22	119.6 (3)
C10—C11—H11A	108.5	C24—C23—H23	120.2
C12—C11—H11B	108.5	C22—C23—H23	120.2
C10—C11—H11B	108.5	C25—C24—C23	121.1 (3)
H11A—C11—H11B	107.5	C25—C24—Br1	119.6 (2)
C13—C12—C11	113.1 (2)	C23—C24—Br1	119.3 (2)
C13—C12—H12A	109.0	C24—C25—C26	119.5 (3)
C11—C12—H12A	109.0	C24—C25—H25	120.3
C13—C12—H12B	109.0	C26—C25—H25	120.3
C11—C12—H12B	109.0	C21—C26—C25	119.9 (3)
H12A—C12—H12B	107.8	C21—C26—H26	120.0
C12—C13—C14	113.6 (2)	C25—C26—H26	120.0
			
O1—C1—C2—C3	−164.4 (2)	C10—S1—C9—C6	174.90 (18)
C6—C1—C2—C3	16.5 (3)	C10—S1—C9—S2	−5.28 (17)
C1—C2—C3—C7	74.2 (3)	C9—S1—C10—C11	−166.51 (17)
C1—C2—C3—C4	−46.7 (3)	S1—C10—C11—C12	−61.2 (2)
C1—C2—C3—C8	−164.2 (2)	C10—C11—C12—C13	−177.7 (2)
C2—C3—C4—C5	55.8 (3)	C11—C12—C13—C14	−177.6 (2)
C7—C3—C4—C5	−65.4 (3)	C12—C13—C14—C15	178.2 (2)
C8—C3—C4—C5	174.0 (2)	C13—C14—C15—C16	176.2 (2)
C21—N1—C5—C6	179.2 (2)	C14—C15—C16—C17	−179.0 (2)
C21—N1—C5—C4	−1.1 (4)	C15—C16—C17—C18	177.6 (2)
C3—C4—C5—N1	144.6 (2)	C16—C17—C18—C19	−176.8 (2)
C3—C4—C5—C6	−35.7 (3)	C17—C18—C19—C20	176.9 (2)
N1—C5—C6—C9	1.9 (4)	C5—N1—C21—C22	65.1 (4)
C4—C5—C6—C9	−177.8 (2)	C5—N1—C21—C26	−117.8 (3)
N1—C5—C6—C1	−178.2 (2)	C26—C21—C22—C23	0.9 (4)
C4—C5—C6—C1	2.1 (3)	N1—C21—C22—C23	177.9 (2)
O1—C1—C6—C5	−171.3 (2)	C21—C22—C23—C24	−0.6 (4)
C2—C1—C6—C5	7.8 (3)	C22—C23—C24—C25	−0.3 (4)
O1—C1—C6—C9	8.6 (3)	C22—C23—C24—Br1	−179.5 (2)
C2—C1—C6—C9	−172.3 (2)	C23—C24—C25—C26	0.9 (4)
C5—C6—C9—S2	4.4 (3)	Br1—C24—C25—C26	−179.9 (2)
C1—C6—C9—S2	−175.45 (17)	C22—C21—C26—C25	−0.3 (4)
C5—C6—C9—S1	−175.79 (18)	N1—C21—C26—C25	−177.4 (2)
C1—C6—C9—S1	4.4 (3)	C24—C25—C26—C21	−0.6 (4)

### Hydrogen-bond geometry (Å, °)

**Table:**

*D*—H···*A*	*D*—H	H···*A*	*D*···*A*	*D*—H···*A*
N1—H1···S2	0.88 (1)	2.11 (2)	2.876 (2)	146 (3)
